# Elective full pulpotomy in mature permanent teeth diagnosed with symptomatic irreversible pulpitis: a two years retrospective study

**DOI:** 10.1007/s00784-024-05814-z

**Published:** 2024-07-08

**Authors:** Cristina Jiménez-Martín, Jenifer Martín-González, Isabel Crespo-Gallardo, Paloma Montero-Miralles, Daniel Cabanillas-Balsera, Juan J. Segura-Egea

**Affiliations:** 1 Department of Stomatology (Endodontic section), School of Dentistry, University of Sevilla, Sevilla, Spain; 2 Facultad de Odontología, Universidad de Sevilla, 41009 Sevilla, Spain

**Keywords:** Calcium silicate–based cement, Caries lesion management, Endodontics, Full pulpotomy, Irreversible pulpitis, Wolters classification of pulpitis

## Abstract

**Aim:**

To investigate the outcome of elective full pulpotomy, using calcium silicate-based cements (CSBC), after 2 years, in symptomatic mature permanent teeth with carious lesions, diagnosed as irreversible pulpitis, and analyse the capacity of Wolters et al. (2017) classification to predict the likelihood of treatment failure.

**Methods:**

The treatment records of 56 patients with symptomatic mature teeth with carious lesions, diagnosed as irreversible pulpitis and treated by elective full pulpotomy, using CSBCs as pulp capping materials, were reviewed. Thirteen teeth were excluded. The remaining 43 teeth were evaluated retrospectively at 24 months. Fisher`s exact test with the Lancaster’s mid-P adjustment was used to assess different outcomes amongst the diagnostic categories.

**Results:**

Four of the cases failed before 24 months and required root canal treatment (RCT). Overall success rate at 2 years was 90.7% (39 of 43). An inverse, but non-significant, correlation was observed between the severity of pulpitis according to the Wolters classification and the treatment success rate (*p* > 0.05). The type of CSBC used was associated to the success rate (OR = 10.5; 95% C.I. = 0.5 – 207.4; *p* = 0.027), being 82% with Endosequence and 100% with Biodentine. Postoperative pain associated significantly to lower success rate (66.7%) (Odds ratio = 8.0; 95% C.I. = 0.7 – 95.9; *p* = 0.047).

**Conclusions:**

Elective full pulpotomy using a CSBC was a successful choice for the treatment of mature permanent teeth with symptoms indicative of irreversible pulpitis. There were no significant differences between the success rate of mild, moderate and severe pulpitis. Postoperative pain could be considered a risk marker for failure of full pulpotomy. The term “irreversible pulpitis” should be re-signified to indicate the need for access to the pulp chamber, rather than an indication for extraction or RCT.

## Introduction

Caries lesion is the most common cause of pulpitis in mature permanent teeth [30]. For decades, pulpitis has been classified, according to the AAE diagnostic criteria [3], as reversible and irreversible. Vital pulp therapy techniques, including pulp capping and pulpotomy, have been used to treat reversible pulpitis [14], while irreversible pulpitis was treated using root canal therapy (RCT) [33].

Lingering pain after cold stimuli and spontaneous pain, are the main criteria used by the AAE classification to determine that pulp is irreversibly affected [3, 4]. Typical pain characteristics that lead to the diagnosis of symptomatic irreversible pulpitis are: sharp and lingering pain triggered by thermal stimulus (often 30 s or longer after stimulus removal), spontaneous pain and often referred pain. The pain can be accentuated by postural changes such as lying down or bending over, and over-the-counter analgesics are typically ineffective [4].

However, numerous studies have shown that there is no exact correlation between the pain symptom and the pulpal inflammatory state [13, 15, 23, 30]. Histological studies have shown that in patients with symptoms of irreversible pulpitis, the inflammatory and infectious process often involved only the coronal pulp tissue underlying the carious lesion, with the root pulp tissue being practically healthy [41]. These findings suggest that strict application of the AAE classification could lead to over-prescription of root canal treatment [17, 33]. Furthermore, in some cases of pulpitis, theoretically irreversible, vital pulp therapy techniques could allow pulpitis to be cured without having to perform RCT. In 2017, Wolters et al. [51] suggested leaving aside the AAE dichotomous classification pulpitis and applying a new classification that recommended more conservative and less invasive treatments. This new classification completely changed the therapeutic attitude towards pulpitis. For Wolters et al. [51] the goal of treatment should be to remove irreversibly inflamed tissue and preserve the remaining normal or reversibly inflamed pulp tissues. The main concept is that vital pulp tissue, managed properly, is quite resistant, and a diseased pulp can heal if most of the inflamed/necrotic tissue is removed [51]. Along these same lines, the ESE clinical practice guideline on the treatment of pulpal and apical disease [26], recommended pulpotomy, full or partial, for patients diagnosed with nontraumatic pulpitis associated with spontaneous pain in permanent teeth. Moreover, pulpotomy is generally quicker, less technically complex, and less invasive than root canal treatment, reducing the risk of unwanted effects such as fracture, or residual periapical inflammation [24, 26].

A full, coronal or complete pulpotomy involves the ‘complete removal of the coronal pulp and the application of a biomaterial onto the pulp tissue at the level of the root canal orifice(s)’ [24]. Numerous studies have been published demonstrating that full pulpotomy, carried out using mineral trioxide aggregate (MTA), or CSBCs such as Biodentine (Septodont) or Endosequence (Brasseler), achieves a success rate greater than 90% in teeth with supposedly irreversible pulpitis symptoms [7, 9, 16, 18, 22, 46–48]. These results have led the ESE to recommend full pulpotomy for patients with teeth diagnosed with nontraumatic pulpitis associated with lingering pain after cold stimuli or spontaneous pain [26].

However, most of the studies on the outcome of full pulpotomy in teeth with irreversible pulpitis do not provide data on some important variables, such as clinical data on which the preoperative diagnosis was based, depth of the carious lesion, and characteristics of the patient's pain. Moreover, although the capability of the Wolters classification in predicting the likelihood of partial pulpotomy failure has been investigated [13], data are needed on its ability to predict the outcome of full pulpotomy. Furthermore, published studies on the outcome of full pulpotomy usually refer to cases in which pulpotomy was performed after the occurrence of pulp exposure during carious tissue removal [31, 36, 39, 46–48]. There have been few studies evaluating the outcome of full pulpotomy performed electively to treat irreversible pulpitis [5, 40].

The aim of this study was to retrospectively investigate the outcome of elective full pulpotomy after 2 years, using CSBC, in symptomatic mature permanent teeth with carious lesions, diagnosed according to AAE classification as symptomatic irreversible pulpitis, and investigating the factors that may affect the outcome of the treatment. As a secondary objective, the capacity of Wolters et al. [51] classification to predict the likelihood of treatment failure was analysed.

## Methods

### Case selection

The Ethics Committee of the institutional review board of the University of Sevilla approved our retrospective study (protocol number 2024–1209). The study was performed in accordance with the ethical standards as laid down in the 1964 Declaration of Helsinki and its later amendments or comparable ethical standards. All patients included in the study had given their informed consent so that data from their medical history could be used in research.

This retrospective study has been conducted according to PROBE 2023 guidelines for reporting observational studies in Endodontics [35]. The study was carried out with the medical records of patients treated from January 2021 to December 2021.

All included patients had been diagnosed as having irreversible pulpitis according to the AAE, so RCT had been indicated. However, if after accessing the pulp chamber the bleeding had been controlled in less than 5 min, elective full pulpotomy had been performed. Before receiving treatment, all patients had given informed consent so that their data could be used in any research study. Clinical data on the patients that had undergone full pulpotomy, including their radiographs, were retrieved from the database.

Fifty-six patients between 11–76 years old diagnosed with irreversible pulpitis [4] and reporting occlusal/proximal caries lesion, whose clinical history included data on the characteristics of the pain and the intraoral examination that justified the diagnosis of irreversible pulpitis, were included.

### Inclusion and exclusion criteria

The inclusion criteria were: (1) pulpotomy had been carried out in mature teeth with a probing pocket depth and mobility within normal limits; (2) with a carious lesion reaching the middle or inner third of dentine [24]; (3) had a preoperative diagnose of irreversible pulpitis according to the AAE classification [4], and providing data about pain characteristics and response to pulp sensitivity tests; (4) had no signs of sinus tract or swelling; (5) periapical radiograph indicated a normal periapical status (PAI score 1–2) [37]; (6) the tooth had been restored; (7) the medical record provided data on the outcome of the treatment over 24 months.

The exclusion criteria were: (1) pulpotomy in immature teeth; (2) pulpotomy carried out after pulp exposure during the removal of carious tissue; (3) pulpotomy performed to treat a pulpal exposure of traumatic origin; (4) the treatment record did not provide sufficient information on the clinical data supporting the diagnosis of irreversible pulpitis; (5) the medical record does not provided information about the outcome of the treatment over 24 months; (6) patients with systemic disease that could affect the outcome of treatment.

### Treatment protocol of pulpotomy

All database treatments had been carried out by the same operator (C.J-M.), which had followed the same protocol in all cases, but using two different CSBC: Biodentine (Septodont) or Endosequence BC RRM Fast Set Putty (Brasseler).

After diagnosis of irreversible pulpitis, local anesthesia had been carried out using 4% articaine with 1:100,000 epinephrine. After rubber dam isolation, the crown of the tooth had been disinfected with 5% NaOCl. Removal of carious tissue had been carried out under sterile water cooling using high-speed tungsten carbide fissure burs, slow-speed round burs, and a spoon excavator. In the periphery of the lesion, the affected tissue had been removed until reaching hard dentin, while in the central area of the lesion, the spoon excavator had been used until reaching soft dentin.

After removing the carious tissue, the pulp chamber had been accessed using a round tungsten carbide bur, and the chamber roof had been completely removed. After pulp exposure, pulp vitality had been confirmed by the presence of bleeding pulp tissue and by the achievement of hemostasis by applying a cotton wool moistened with 5% NaOCl for 2 min, with a dry cotton ball at the top. This operation had been repeated if necessary for a period of 5 min [24, 26]. In all cases included in the study, hemostasis had been achieved in less than 5 min. When this had not been achieved, root canal treatment had been prescribed.

Once hemostasis was achieved, the coronal pulp was removed up to cement-enamel junction in single-rooted teeth, and up to the level of root canal orifices in multi-rooted teeth, using a sterile diamond round bur under water coolant and amputated with a sharp spoon excavator. Remains of pulp tissue were removed using the Start-X #1 ultrasonic tip (DentsplySirona). Then, the cavity was flushed with sterile saline water and the pulp wound was stanched with sterile cotton pellet soaked with 5% sodium hypochlorite for 1 min.

A 2-mm-thick layer of CSBC was then applied on the top of the remaining root pulp with gentle pressure with a clean cotton pellet. In multi-rooted teeth, the cement was placed on the root canal orifices and the whole pulp chamber floor following the manufacturer's instructions. After waiting 12 min (Biodentine) or 20 min (Endosequence) for the biomaterial to set, a layer of glass ionomer (Vitrebond Plus, 3 M) was placed. The pulp chamber was rinsed with alcohol to clean any remaining biomaterial, selectively etched with 37% orthophosphoric acid for 30 s, rinsed with water, and dried with the air/water syringe. The composite resin had been applied with the incremental technique and each layer had been polymerized for 20 – 40 s. Once the tooth reconstruction was completed, the occlusion was checked and the restoration was polished. Finally, an end-of-treatment x-ray had been taken and the patient had been scheduled for subsequent check-ups. When an inlay and/or crown was indicated, the patient was rescheduled for a second appointment for the prosthetic restoration.

### Data collection

In each medical history the following data had been recovered: name, age, sex, tooth type, characteristics of pain, probing pocket depth, tooth mobility, interpretations of preoperative radiographs, tooth surfaces affected by caries, depth of the caries lesion, preoperative PAI score [37], and pulpitis diagnose according to AAE classification [3, 4], which in all cases was irreversible pulpitis. The depth of the carious lesion was evaluated on the periapical radiograph and was classified into two types, depending on whether it reached the inner third or the middle third of the dentin.

With the data from the clinical history, each case was classified as mild, moderate or severe pulpitis, according to the classification suggested by Wolters et al. [51].

The following data regarding treatment were also recovered: CSBC used, number of visits and type of the coronal restoration (resin composites or inlay).

Postoperative pain was considered present when patients consulted for pain during the week following treatment. If there was no consultation due to post-treatment discomfort or pain, it was considered absent.

Finally, regarding the postoperative period and follow-up visits, the following data, when they appeared registered, were collected: history of spontaneous pain or discomfort, response to the cold pulp sensitivity test, pain when biting, pain on percussion, probing pocket depth, color changes, radiographic periapical status (PAI score), presence of root canal obliteration, and presence of root resorption.

### Assessment of treatment outcome

The clinical criteria used to assess the outcome of the treatment were based on previous research [25, 46, 53]: no history of spontaneous pain or discomfort, except for the first week after treatment; no swelling; no tenderness to palpation or percussion and the tooth is functional; normal mobility and probing pocket depth; soft tissues around the tooth are normal with no swelling or sinus tract.

In addition, the outcome was also assessed radiographically using the following criteria: absence of emerging root periapical or periradicular radiolucent image, and PAI score ≤ 2. When all these clinical and radiographical conditions were met, the treatment was considered successful.

### Statistical analysis

Sample size was calculated based on the success rate of previous studies [39, 44] using a method of comparing two proportions [42] for a study with α = 5% and power of 80%, a minimum of 42 participants were required for the results to be statistically meaningful.

The collected data was entered into Excel software, Version 2019 (Microsoft). The success rate were determined. Fisher`s exact test with the Lancaster’s mid-P adjustment [11] was used to assess the influence of various factors for pulpotomy success. Haldane-Anscombe correction was used to calculate odds ratios if any of the cell expectations would cause a division by zero error. The OpenEpi Module for Diagnostic Test Evaluation (https://www.openepi.com/DiagnosticTest/DiagnosticTest.htm) [19] was used to analyse postoperative pain as diagnostic indicator for the outcome of pulpotomy. Significance was set at p < 0.05.

## Results

### Sample characteristics

**Fig. 1:**
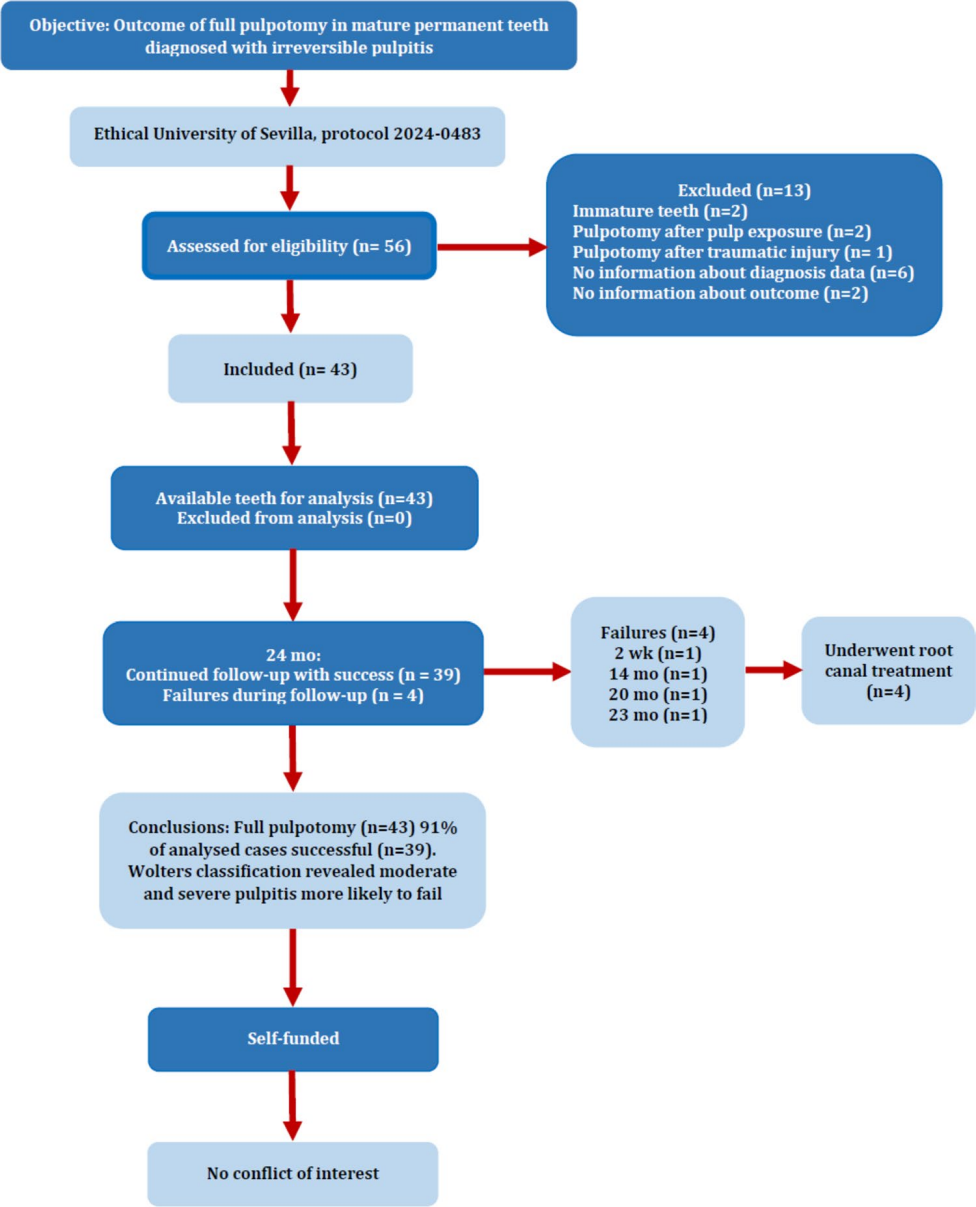
Study flowchart

Initially, 56 pulpotomy cases were found that seemed to meet the inclusion criteria. However, after carefully reviewing the medical records, thirteen cases were excluded for the following reasons: immature teeth (n = 2), pulpotomy after pulp exposure (n = 2), pulpotomy after traumatic injury (n = 1), insufficient data about pain characteristics and diagnosis (n = 6), and no information about the outcome of the treatment over 24 months (n = 2). There remained 43 pulpotomy cases that were included in the study and analysed (Fig. 1).

**Fig. 2:**
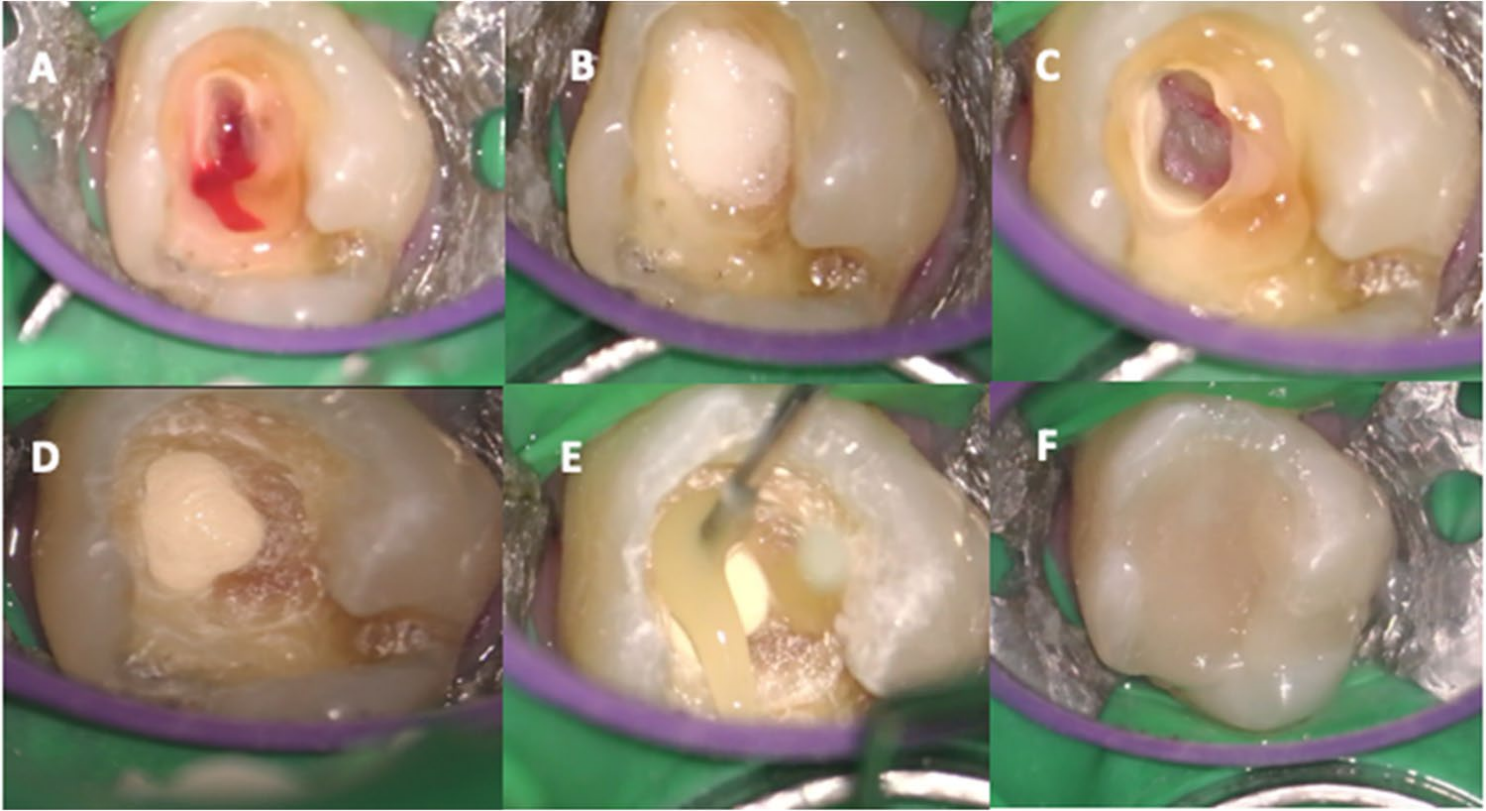
Full pulpotomy clinical protocol. (**A**) After cavity access, coronal pulp tissue was removed. Pulpal hemorrhage is evident. (**B**) Cotton pellet soaked in 2.5% NaOCl, pressed against the exposed pulp tissue. (**C**) Hemostasis obtained after five minutes by placing cotton pellet. (**D**) Biodentine™ used to restore the entire cavity. (**E**) Vitrebond placed between Biodentine and composite material. (**F**) Tooth permanently restored with resin-based composite material

The treatment of all pulpotomies had been carried out by the same operator. The only variation in the protocol had been the type of CSBC: Biodentine (Septodont) had been used in 21 cases and Endosequence (Brasseler) in 22 cases (Fig. 2).

**Fig. 3:**
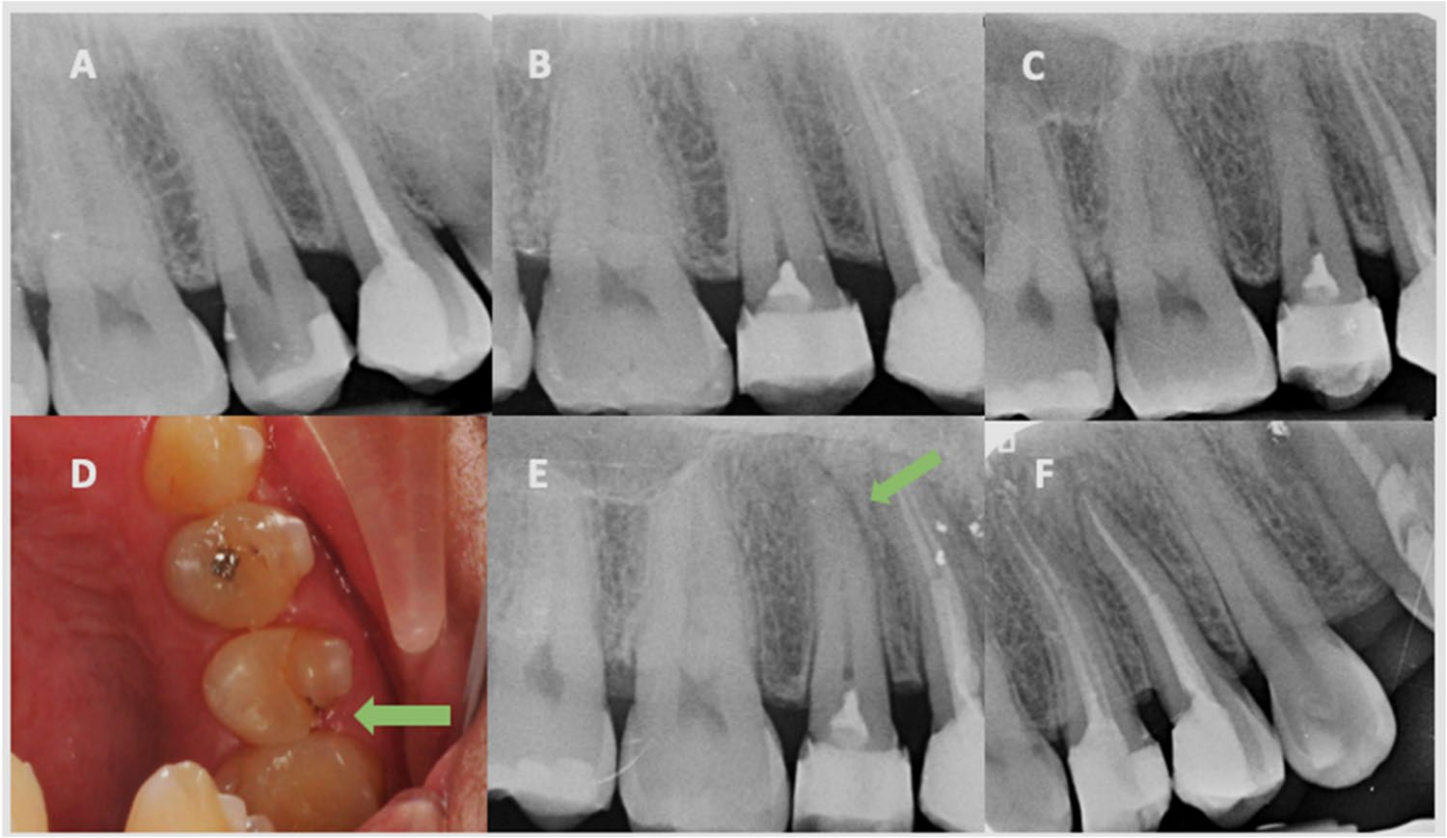
Case that failed after 20 months. (**A**) Right upper second premolar with symptoms of irreversible pulpitis. (**B**) Full pulpotomy with Endosequence. (**C**) Annual review. (**D** & **E**) Patient presents with pain on percussion and leaky composite at 20 months. (**F**) Root canal treatment was performed

The summary outcomes during the follow-up period are shown in Table 1. Four of the cases failed before 24 months and required root canal treatment (RCT). The first failed case had been diagnosed as moderate pulpitis, showing spontaneous pain, pain on biting and tenderness to percussion at 2 weeks, being diagnosed as severe pulpitis and emerging apical periodontitis. RCT was indicated. The next three failures occurred in teeth that had been diagnosed as moderate pulpitis. They occurred at 14, 20 and 23 months, all of them showing intense spontaneous pain and being diagnosed as severe pulpitis. In one of them the restoration was leaked (Fig. 3).

**Table 1:** Summary outcomes during the follow-up period

Follow-up period No. failures	Success rate No (%)	Reason for failure	Previous diagnosis (Wolters) Actual diagnosis (Wolters)	Treatment plan
2 weeks 1 failure	42 of 43 (97.7)	Spontaneous pain; pain on biting; tenderness to percussion	Severe pulpitis Severe pulpitis with emerging apical periodontitis	Root canal treatment
14 months 1 failure	41 of 43 (95.3)	Spontaneous pain	Moderate pulpitis Severe pulpitis	Root canal treatment
20 months 1 failure	40 of 43 (93.0)	Spontaneous pain; leaky composite filling	Moderate pulpitis Severe pulpitis	Root canal treatment
23 months 1 failure	39 of 43 (90.7)	Spontaneous pain	Moderate pulpitis Severe pulpitis	Root canal treatment
24 months 0 failure	39 of 43 (90.7)	–-	–-	–-

### Outcome

The characteristics of the included cases according to the type of pulpitis diagnosed, and the success rates at two years are shown in Table 2. Of the 43 cases with irreversible pulpitis treated with full pulpotomy, 39 met the success criteria after 2 years of follow-up (success rate = 90.7%).

**Table 2:** Record of clinical characteristics and evolution after 2 years follow-up according to the type of pulpitis diagnosed

Classification (Cases)	Pulpitis (Cases)	Age	Teeth	Pain	Depth of caries lesion	Outcome at two years
AAE (n = 43)	Irreversible (n = 43)	28.0 ± 15.4	Single-rooted (n = 9) Multi-rooted (n = 34)	Only provoked = 40 (93%) Spontaneous = 3 (7%)	Middle third = 13 (30%) Inner third = 30 (70%)	4 failures 39 success (90.7%)
Wolters et al. 2017	Mild (n = 12)	26.1 ± 14.9	Single-rooted (n = 1) Multi-rooted (n = 11)	Only provoked = 12 (100%)	Middle third = 11 (92%) Inner third = 1 (8%)	0 failures 12 success (100%)
	Moderate (n = 26)	27.4 ± 15.0	Single-rooted (n = 7) Multi-rooted (n = 19)	Only provoked = 26 (100%)	Middle third = 1 (4%) Inner thrid = 25 (96%)	3 failures 23 success (88.5%)
	Severe (n = 5)	32.4 ± 20.8	Single-rooted (n = 1) Multi-rooted (n = 4)	Only provoked = 2 (93%) Spontaneous = 3 (60%)	Middle third = 1 (20%) Inner third = 4 (80%)	1 failure 4 success (80.0%)

Relating outcome to preoperative diagnosis according to the criteria of Wolters et al. [51] revealed that teeth with mild pulpitis were 100% successful, 88.5% moderate pulpitis successful and severe pulpitis 80.0% successful at 2 year. There were no significant differences between the success rate of “mild” and “moderate” pulpitis (*p* = 0.27), “moderate” and “severe” pulpitis (*p* = 0.68) or “mild” and “severe” pulpitis (*p* = 0.16) at 2 years follow-up.

Table 3 shows the characteristics of the cases and their relationship with the treatment outcome. Sex, age, or the number of roots of the treated tooth did not influence the success rate (*p* > 0.05). Neither the type of preoperative pain, nor the affected tooth surface, nor its depth, significantly influenced the treatment result (*p* > 0.05).

**Table 3:** Preoperative, intraoperative and postoperative factors and their effect on the treatment outcome of pulpotomy after 24-month follow-up

Characteristic	Success rate No. (%)	Odds ratio (95% C.I.)	*p* value * (two-tailed)
Sex Male Female	15 of 17 (88.2) 24 of 26 (92.3)	1.6 (0.2 – 16.6)	0.68
Age (y) ≤ 40 > 40	31 of 34 (91.2) 8 of 9 (88.9)	0.8 (0.1 – 22.8)	0.81
Tooth type Single-rooted Multi-rooted	8 of 9 (88.9) 31 of 34 (91.2)	1.3 (0.0 – 13.8)	0.81
Affected tooth surfaces Approximal with/without occlusal Only occlusal	28 of 32 (87.5) 11 of 11 (100)	3.6 (0.2 – 73.0)	0.19
Depth of carious lesion Inner third Middle third	26 of 30 (86.7) 13 of 13 (100)	4.6 (0.2 – 91.6)	0.14
Spontaneous pain Present Absent	4 of 5 (80.0) 35 of 38 (92.5)	2.8 (0.1 – 34.2)	0.46
Calcium silicate-based cement Endosequence Biodentine	18 of 22 (81.9) 21 of 21 (100)	10.5 (0.5 – 207.4)	0.027*
Appointments / Restoration type Two visits / Inlay One visit / Composite	4 of 5 (80.0) 35 of 38 (92.1)	2.8 (0.1 – 34.2)	0.46
Postoperative pain Present Absent	4 of 6 (66.7) 35 of 37 (94.6)	8.0 (0.7 – 95.9)	0.047**
Marginal leakage Present Absent	10 of 11 (90.9) 29 of 32 (90.6)	1.0 (0.1 – 10.4)	0.98

*Fisher´s exact test with Lancaster's Mid-P adjustment (two-tailed)

**Fisher´s exact test with Lancaster's Mid-P adjustment (one-tailed)

**Fig. 4:**
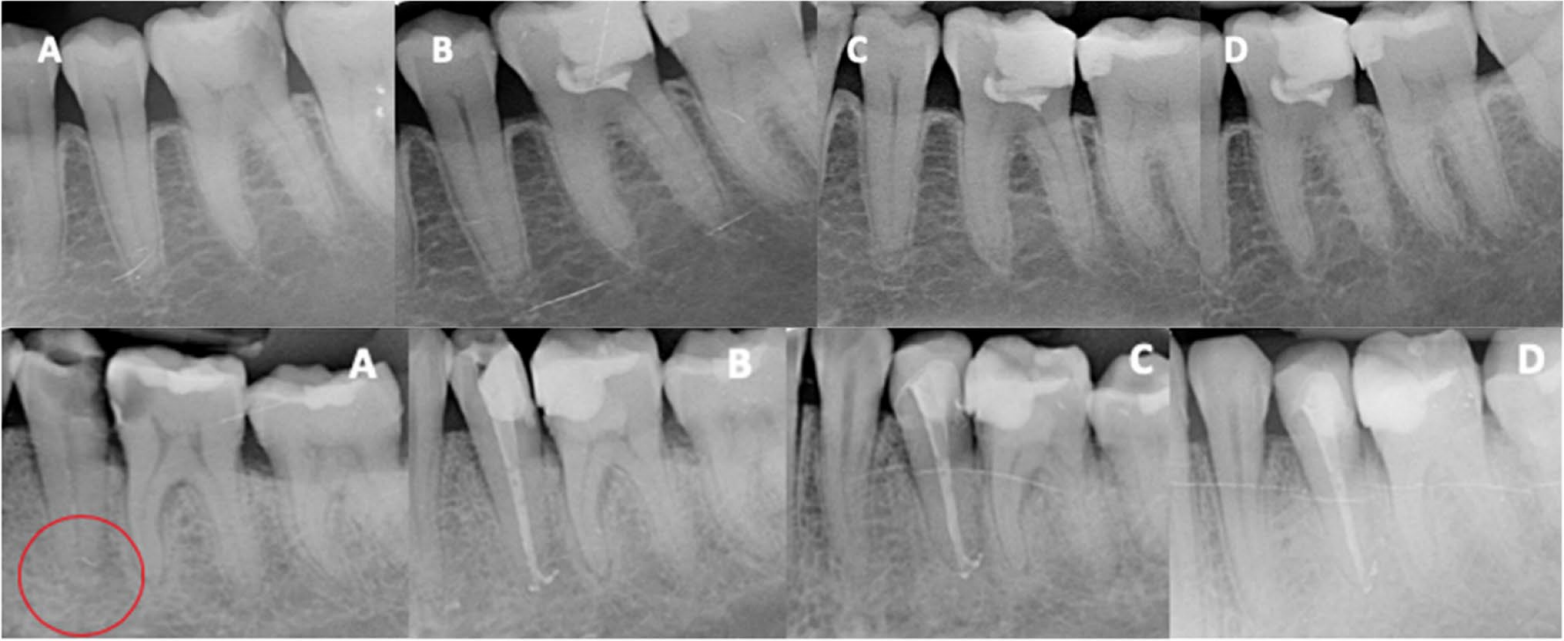
Top: full pulpotomy using Endosequence (**A**) Left lower first molar (36) with symptoms of irreversible pulpitis. (**B**) Full pulpotomy with Endosequence (this material is more radiopaque than biodentine). (**C**) Follow-up x-ray at 12 months. (**D**) Follow-up x-ray at 2-years. Bottom: full pulpotomy using Biodentine. (**A**) Left lower first molar (36) with symptoms of irreversible pulpitis and left lower second premolar (35) with pain on percussion, negative response to pulp test, and signs of apical periodontitis. (**B**) Full pulpotomy with Biodentine (36) and root canal treatment (35). (**C**) Follow-up x-ray at 12 months. (**D**) Follow-up x-ray at 2 years

**Fig. 5:**
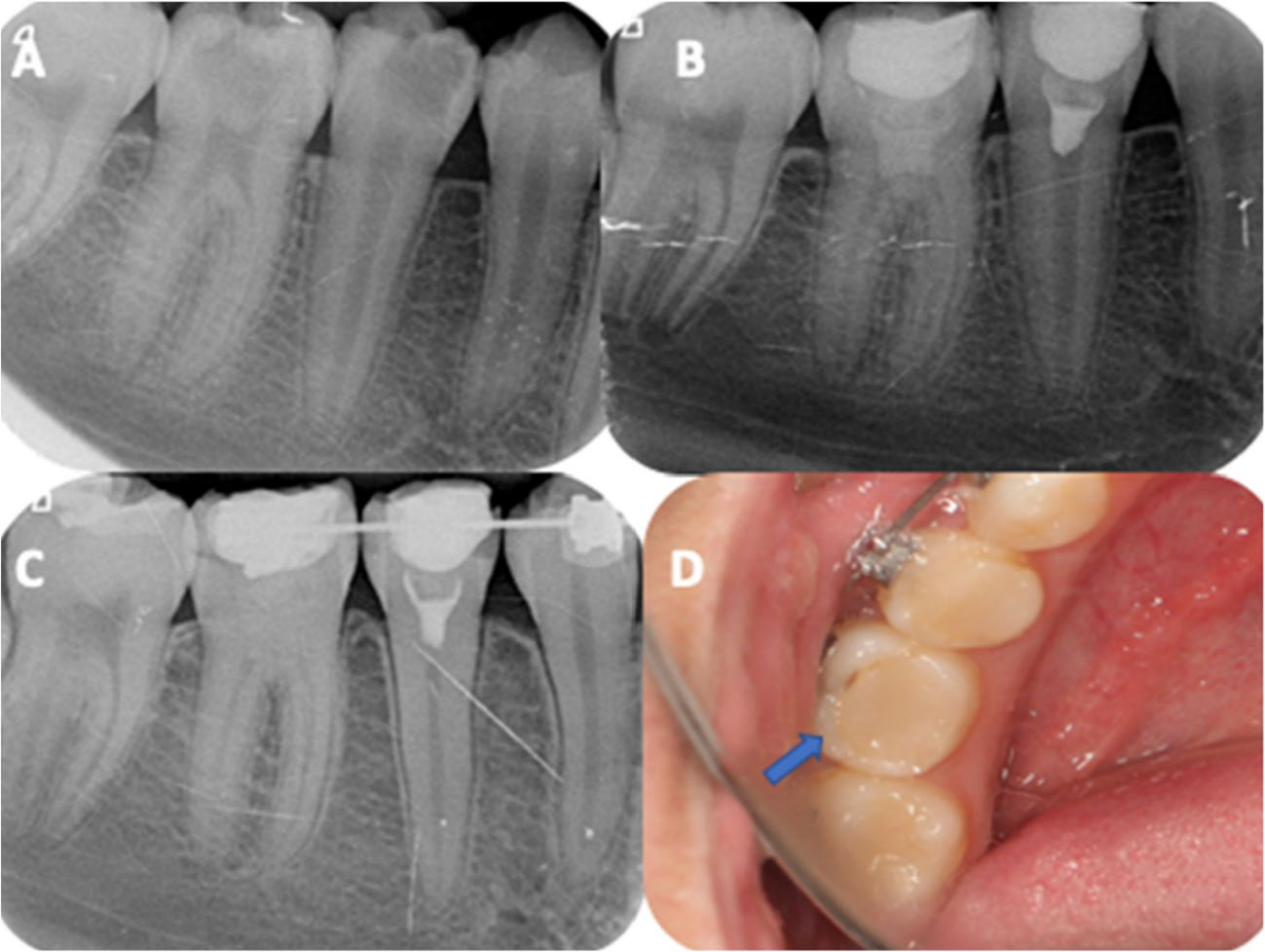
(**A**) Lower right second premolar (45) and first molar (46) with symptoms of irreversible pulpitis. (**B**) Full pulpotomy in 46 using Biodentine, and full pulpotomy in 45 using Endosequence. (**C**) Follow-up x-ray at 2-years months. (**D**) Leaky composite in 46 at 2-years follow-up; patient was asymptomatic

Regarding intraoperative factors, bleeding time after pulp access had been ≤ 5 min in all cases, without the medical records collecting more details. No significant association was found with the type of restoration performed and the number of appointments (*p* > 0.05). On the contrary, the type of CSBC used was associated to the success rate (OR = 10.5; 95% C.I. = 0.5 – 207.4; *p* = 0.027). In the treatments performed with Endosequence there were 4 failures (success rate of 81.9%), while in those performed with Biodentine there were no failures (100% success) (Figs. 4 and 5).

Marginal leakage of restoration was not related to treatment outcome (p > 0.05). Only one of the four failure cases showed marginal leakage. However, a significant association was found between the presence of postoperative pain and the treatment outcome.

Cases in which the patient had postoperative pain (14.0%) showed a significantly lower success rate (66.7%) than cases in which there was no postoperative pain (95.9%) (Odds ratio = 8.0; 95% C.I. = 0.7 – 95.9; p = 0.047). Presence of postoperative pain showed a sensitivity of 50% (15.0%—85.0%), a specificity of 89.7% (76.4%—95.9%) and a diagnostic accuracy of 86.1% (72.7%—93.4%). Its negative predictive value was 94.6% (82.3%—98.5%). No correlation was found between postoperative and preoperative pain (p = 0.68).

None of the successfully treated teeth showed pain on biting, pain on percussion, abnormal probing pocket depth, presence of root canal obliteration, or presence of root resorption throughout the follow-up period.

At 24 months, the 39 teeth evaluated showed PAI scores less than or equal to 2. Only four teeth (10.3%) responded positively to the cold pulp sensitivity test: an upper second premolar (15), two lower first molars (36 and 46), and one upper first molar (26). Likewise, in only one of the treated teeth (45) a darkening of the crown was observed.

## Discussion

This retrospective study aimed, first of all, to analyze the outcome of elective full pulpotomy in mature permanent teeth with carious lesions and diagnosed as symptomatic irreversible pulpitis, after 2 years of follow-up. The results have shown a success rate of 90.7%, indicating that elective full pulpotomy is a successful treatment for irreversible pulpitis in mature permanent teeth with carious lesions. These results, together with those of a large body of previous research [34], show that full pulpotomy is effective and predictable, and can be considered a simpler, less invasive and cheaper option to RCT for the management of irreversible pulpitis in mature teeth.

Regarding the second objective, to analyse the ability of Wolters classification [51] to predict failure of full pulpotomy, the results show an inverse correlation between the severity of pulpitis and the success rate of the treatment, with the success rate being 100% and 88.5% in cases diagnosed as mild or moderate pulpitis, respectively, observing the lowest success rate (80%) in cases of severe pulpitis. However, these differences were not statistically significant (*p* > 0.05).

The methodology used in this study has followed the PROBE 2023 guidelines for reporting observational studies in Endodontics [35]. Although it is a retrospective study, it is important to note that all the included cases had been performed by the same operator, who had followed the same strict protocol in all of them. The only intraoperative variable had been the type of CSBC. Likewise, th operator had taken very precise notes of the variables analyzed, which subsequently facilitated data collection and avoided many of the drawbacks that most retrospective studies have. Moreover, in all cases included in the study, the clinical history stated that pulpal hemostasis had been achieved in less than 5 min, suggesting that the pulp inflammation was limited in all cases to the coronal pulp [1]. Then, full pulpotomy had been well indicated according to scientific evidence [24, 26].

It is important to make some comments on the statistical treatment carried out. The Lancaster's mid-P (LMP) correction to the Fisher’s exact test (FET) has been used. The conventional approach to calculating the p value for FET has been shown to be conservative, requiring more evidence than is necessary to reject a false null hypothesis [11]. On the contrary, the LMP correction to the FET is less conservative and more powerful. The LMP test provide a better balance of Type I and Type II errors, when compared with the FET, and produce higher levels of power than the FET while still adhering closely to the desired 0.05 significance level.

Full pulpotomy has been proposed as an alternative to RCT for the management of teeth with deep carious lesions and symptoms of irreversible pulpitis [24, 26]. In the present study, unlike in others published previously [31, 36, 39, 46–48], full pulpotomy was not performed after pulp exposure following the removal of carious tissue, but was directly chosen, after the patient's informed consent, as the elective treatment for symptomatic irreversible pulpitis. Other studies had already analyzed the results of elective full pulpotomy in relation to the success rate [46, 53] or the response to pulp tests in the treated teeth [5], with alike results. Taha et al. [46] and Zhu et al. [53] found a success rates at 12-months of 97.5% and 92.6%, respectively, for elective full pulpotomy.

The success criteria that have been applied to assess the outcome of the treatment are similar to those previously used by other researchers [25, 46, 53]. The success rate calculated is comparable to that found in other retrospective studies, such as 93.1% [36] and 92.6% [53]. The slightly lower value may be due to the fact that both previous studies only had a 12-month follow-up, while the present study has a 24-month follow-up. However, the success rates found in previous prospective studies [9, 38, 40] and in randomized clinical trials [31, 39, 48], including teeth with symptomatic irreversible pulpitis, are also very similar, ranging 89.8%-93.8%.

Among all the variables analyzed, sex, age and the number of roots did not influence the success rate. Other previous studies have also not found any influence of these factors on the outcome of full pulpotomy [47]. Furthermore, the reviews carried out [25, 28] have concluded that the result of partial/complete pulpotomy is not influenced by age and can be considered a successful treatment in men and women of any age, and in any type of tooth.

The depth of the carious lesion has been related to both the preoperative state of the pulp and the outcome of the treatment [25, 30]. Moreover, the result of partial pulpotomy has been shown to be influenced by the depth of the carious lesion [13]. However, in the present study, although teeth with deep carious lesions had a lower success rate (87%) compared to those that did not reach the inner third (100%), there was no significant association between the caries depth and the success rate (*p* > 0.05). This could be because in neither case the carious lesion was extremely deep. Only 30 of the included cases were deep carious lesions [24], reaching the inner third of the dentin, while the other 13 were lesions that only reached the middle third. It has been shown that when the lesion affects the inner quarter of the dentin, the bacteria predominate in the primary dentin [12], and only contact the pulp in extremely deep lesions, in which inflammatory infiltrate and partial pulp necrosis are found underlying the lesion [20].

The location of the caries lesion and the affected surface did not significantly influence the treatment result (*p* > 0.05), although the success rate was lower (87%) in patients with involvement of the proximal surfaces. Other studies have found similar findings [36, 40]. Nor has any influence of the affected surface on the postoperative response to cold stimuli been found [5].

It could be expected that the type of postoperative pain, spontaneous or provoked, would influence the prognosis of the treatment, since it would supposedly correlate with the severity of the pulpitis. However, in the present study, although the presence of spontaneous preoperative pain was correlated with a lower percentage of success (80%), compared to patients who only showed provoked pain (92.5%), the difference was not significant (*p* > 0.05). Since spontaneous pain determines the diagnosis of severe pulpitis, according to Wolters et al. classification [51], the same success rate was found in cases of severe pulpitis. No significant correlation was found between the diagnosis according to Wolters et al. [51] classification and the treatment outcome, but the success rate decreased with the severity of the pulpitis. Other study has found significantly less successful outcome in cases of partial pulpotomy with severe pulpitis, compared with mild pulpitis [13]. Studies with larger samples are necessary to definitively determine the correlation between the diagnosis according to Wolters and the outcome of treatment.

Several previous studies have found a relationship between the type of restoration and the result of the treatment [21, 36, 49], but in the present study no influence of the type of restoration has been found. This result is in accordance with that of a systematic review [2] finding that the type of restoration did not significantly affect the success rates of full pulpotomy.

The results of the study that has been carried out show only two factors that significantly influenced the treatment outcome: the type of CSBC and postoperative pain. All four cases of failure occurred in patients in whom Endosequence had been used as a pulp capping material, with a success rate of 82%. However, all cases treated with Biodentine were successful. These results are in accordance with the findings of previous studies. Those in which Biodentine was used, provided success rates greater than 90% [10, 31, 45, 47]. Endosequence has been less investigate as pulp capping material in full pulpotomy, obtaining success rates at 12-months of 93.3% [32] and 77.5% [22].

Regarding the postoperative pain, it was present only in 14% of patients. While postoperative pain is less in pulpotomy than in RCT [29], postoperative pain affect 27%-82% of patients who have undergone full pulpotomy [7, 43]. Taking into account that presence of preoperative pain is the major predictor of postoperative pain [50], the low percentage of patients who felt postoperative pain in the present study may be due to the also low percentage of patients who showed spontaneous preoperative pain. Eghbal et al. [27] observed complete remission of symptoms 24 h following full pulpotomy.

Although the presence of postoperative pain did not correlate with preoperative pain, it was strongly associated to lower success rate of full pupotomy (67%) (Odds ratio = 8.0; 95% C.I. = 0.7 – 95.9; *p* = 0.047). The ability of postoperative pain to discriminate between cases that will have a good prognosis and those that will fail is high, with a prognostic accuracy of 86%. However, the relationship between postoperative pain and the outcome of full pulpotomy should be analyzed in new studies.

Concerning the response to pulp sensitivity tests, only 10% of cases responded positively to the cold test. This result agrees with what other authors have reported [5]. Well, it is normal to find that teeth with full pulpotomy are unresponsive [24].

Vital pulp therapy procedures, such as full pulpotomy, should be assessed 6 and 12 months postoperatively and at yearly intervals (if necessary) for 4 years thereafter [24]. The follow-up period of the present study, 24-months, is longer than that of other previously published studies [36, 39, 40, 46–48, 52, 53], what should be considered a strength. On the contrary, this study has some limitations. Most of the clinical studies carried out to analyze the outcome of full pulpotomy include cases in which treatment was indicated after pulp exposure occurred during caries removal [31, 36, 39, 46, 48]. On the contrary, there are few studies in which, as in the present one, full pulpotomy was performed electively to treat cases diagnosed as irreversible pulpitis [5, 40, 53]. The comparison of the success rates of both types of studies could be criticized, since the indication for treatment is substantially different. However, in the present study, the removal of carious tissue was carried out following the selective removal protocol, without opening the chamber until the carious tissue had been completely removed. On the other hand, the available evidence indicates that the type of pulp exposure treated does not affect the results of pulpotomy [25].

**Fig. 6:**
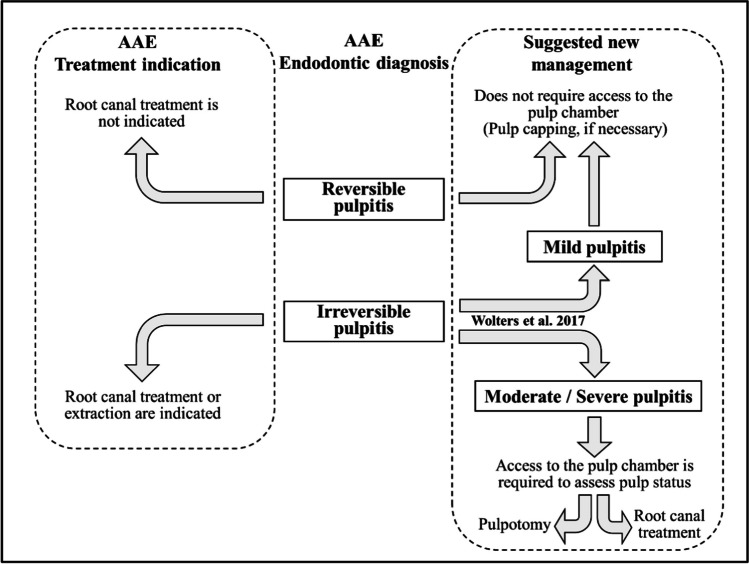
Proposal for a new interpretation of the term "irreversible" as indicative of the need to access the pulp chamber to analyze the pulp state and decide accordingly

For many years, the AAE criteria [3] on the treatment of irreversible pulpitis led the dentist to ask the patient the choice between RCT and extraction. Without a doubt, this way of proceeding has led to the extraction of a large number of teeth that could have been kept in the mouth using VPT techniques [17, 33]. Uninsured patients, low-income patients, and patients with limited access to specialty care often opted for the extraction of restorable teeth with irreversible pulpitis [8]. In view of the present results, and those of all the clinical studies referenced above, the term “irreversible” can no longer be understood in the sense that the AAE has been using it [6], i.e. that the management of the affected tooth requires RCT or extraction [4]. On the contrary, the diagnosis of "irreversible pulpitis" should be understood as requiring access to the pulp chamber to assess the pulp status and to decide the extent of treatment (pulpotomy or RCT) (Fig. 6), according to what was proposed by Wolters et al. [51].

## Conclusions

Elective full pulpotomy using CSBC was a successful choice for the treatment of mature permanent teeth with carious lesions originating symptoms indicative of irreversible pulpitis. It is simpler, less invasive and cheaper than root canal treatment. Postoperative pain could be considered a risk marker for failure of full pulpotomy.

An inverse, but non-significant, correlation was observed between the severity of pulpitis according to the Wolters classification and the treatment success rate. The present results, together with the large amount of scientific evidence accumulated in recent years demonstrating that the erroneously called *irreversible* pulpitis can be treated by maintaining, totally or partially, pulp vitality, highlights the need to definitively discard the conventional terminology of *irreversible pulpitis*,or maintain it but modifying its meaning towards the need to access the pulp chamber.
